# 

**Published:** 1915-05-01

**Authors:** 


### CONTENTS.

| | PAGE |
|---|---|
| **Military Medical Rank and Civil Practice** ... | 99 |
| **The War Work of the Voluntary Hospitals** ... | 100 |
| **Hospital and Institutional News** ... ... ... | 101 |

The King at the Great Northern Hospital—The Woman Hospital Orderly—King Albert's Hospital for Convalescent Belgian Soldiers—Central Stores for Poor-Law Infirmaries—An Emergency Measure—From Hospital Foreman to Chairman of the Guardians—Leicester as a Hospital Centre—An Extraordinarily Belated Report—A Cottage Hospital and the War Office—Lewisham Infirmary as a Military Hospital—An Atmosphere of Economic Research—Woman on Her Own—The Indifferent Testator—A Projected Cottage Hospital for Coalville—The Bed Problem at Derby—Montenegro and Sanitation—The Sight of the Blind—Worcester General Infirmary—The London School Medical Service—The Sale of Narcotic Drugs—The War Office and the Private House—The Fate of the Circular—Chairman of West Ham Hospital Resigns—The Home-Made Respirator—Colour in the Annual Report—This Week's Drug Market.

| | PAGE |
|---|---|
| **Eye Injuries in Warfare** ... ... ... ... | 107 |
| The Reports at the Ophthalmological Congress. | |
| **Buildings Contemplated** ... ... ... ... | 1C8 |
| **The Standard of Mental Administration** ... | 109 |
| Sir Thomas Clouston's Theories and Practice. | |
| **Treatment by Organic Extracts** ... ... ... | 110 |
| A Fatal Case. | |
| **A Medical Causerie** ... ... ... ... ... | 111 |
| **Round the World** ... ... ... ... ... | 112 |
| Fellow-Workers and the Roll of Honour. | |

[*See over.*]

### CONTENTS—(*continued*).

| | PAGE |
|---|---|
| **Medical Treatment of Soldiers on Sick Leave** | 113 |
| A Plea for Co-operation between the War Office and the National Insurance Commissioners. | |
| **Legal Notes for Institutional Workers** ... | 114 |
| The Insurance Commissioners' Regulations Challenged. | |
| Capacity for Light Work. | |
| **The Institutional Library** ... ... ... ... | 115 |
| The House Surgeon's Art. | |
| On Morbid Cycles. | |
| Middle Age and Moderation. | |
| Martindale and Westcott. | |
| Christian Science. | |
| **The Book Market** ... ... ... ... ... | 116 |
| **Personalia** ... ... ... ... ... ... | 117 |
| **The Editor's Letter-Box** ... ... ... ... | 117 |
| The Welfare of Blinded Soldiers. | |
| The After-Care of the Wounded. | |
| More Doctors Wanted—A Suggestion. | |
| **Remarkable Hospital Chapels** ... ... ... | 118 |
| Chapel of Great Barr Hall Institution, near Birmingham. | |
| **Admiralty Doctors** ... ... ... ... ... | 118 |
| **The Institutional Worker** ... ... (Suppl.) | 1 |
| V.—The Pharmacist. | |
| Question for May. | |
| Questions Invited. | |
| Enquiries and Answers. | |
| Employment and Training. | |
| Miscellaneous. | |

